# The Genus* Phyllanthus*: An Ethnopharmacological, Phytochemical, and Pharmacological Review

**DOI:** 10.1155/2016/7584952

**Published:** 2016-04-20

**Authors:** Xin Mao, Ling-Fang Wu, Hong-Ling Guo, Wen-Jing Chen, Ya-Ping Cui, Qi Qi, Shi Li, Wen-Yi Liang, Guang-Hui Yang, Yan-Yan Shao, Dan Zhu, Gai-Mei She, Yun You, Lan-Zhen Zhang

**Affiliations:** ^1^School of Chinese Materia Medica, Beijing University of Chinese Medicine, Beijing 100102, China; ^2^Institute of Chinese Materia Medica, China Academy of Chinese Medical Sciences, Beijing 100700, China; ^3^Institute of Zoology, Chinese Academy of Sciences, Beijing 100101, China; ^4^Key laboratory of Chinese Internal Medicine, Beijing University of Chinese Medicine, Beijing 100700, China

## Abstract

The plants of the genus* Phyllanthus* (Euphorbiaceae) have been used as traditional medicinal materials for a long time in China, India, Brazil, and the Southeast Asian countries. They can be used for the treatment of digestive disease, jaundice, and renal calculus. This review discusses the ethnopharmacological, phytochemical, and pharmacological studies of* Phyllanthus* over the past few decades. More than 510 compounds have been isolated, the majority of which are lignins, triterpenoids, flavonoids, and tannins. The researches of their remarkable antiviral, antioxidant, antidiabetic, and anticancer activities have become hot topics. More pharmacological screenings and phytochemical investigations are required to support the traditional uses and develop leading compounds.

## 1. Introduction

*Phyllanthus* (Euphorbiaceae) is a large genus and widely distributed in tropical and subtropical zones like tropical Africa, tropical America, Asia, and Oceania. This genus, consisting of more than 700 species, can be classified into 11 subgenuses [1, 2]. The most popular 24 species are chiefly belonging to subgenus* Kirganelia*,* Cicca*, and* Phyllanthus* and they are traditionally used by different nationalities.

Genus* Phyllanthus* has been employed as herbal drugs for a long time in China, India, Brazil, and Southeast Asian countries. The most abundant species are used in India and have a beneficial role in Ayurveda for the treatment of digestive, genitourinary, respiratory, and skin diseases [3, 4]. In China, herbs and their prescriptions are used to treat hepatitis B, hypertension, dropsy, and sore throat [2]. These herbal drugs are employed by local inhabitants of Thailand, Latin America (especially Brazil), and Africa to cure jaundice, renal calculus, and malaria, respectively [5–7].

By virtue of the wide uses of* Phyllanthus* as anti-HIV, anticancer, and anti-HBV agents, there has been considerable interest in the investigations of this genus in recent years and the researches about pharmacology and chemistry had been finished in a deep going way. This report reviews the ethnopharmacological, phytochemical, and pharmacological investigations of* Phyllanthus* over the past few decades. More than three hundred articles were selected from the data taken from SciFinder Scholar database by searching the keyword “*Phyllanthus*”.

## 2. Ethnopharmacological Uses

**Figure 1:**
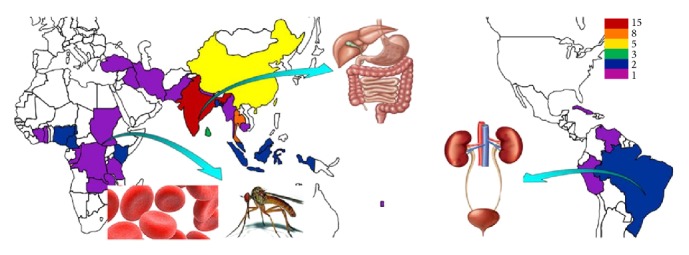
Traditional use of genus* Phyllanthus* in different countries. Different color represents the number of plants traditionally used in different countries: red, orange, yellow, green, blue, and purple represent fifteen, eight, five, three, two, and one kinds of plants under use, respectively. In Asia,* Phyllanthus* are used to treat digestive system disease, in south America,* Phyllanthus* are used to treat urinary system disease, and in Africa,* Phyllanthus* are used to treat malaria and wound.

The traditional application experiences of these herbs may have reference value for the treatment of recent diseases. Botanical data, folk name, and medicinal properties of twenty-four* Phyllanthus* species are depicted in Table 1. In Asia, seventeen plants are considered to have bitter and astringent taste. They are regarded as stomachic, diuretic, febrifuge, deobstruent, and antiseptic agents and effective remedies for hepatopathy, hypertensive, diabetes, and jaundice. In Africa, six herbs are widely employed by many tribes for the treatment of malaria wound and tetanus. Six species are used extensively in Latin America for the treatment of urination disorder and diabetes. The distribution and the main uses of* Phyllanthus* are pictured in Figure 1.

### 2.1. Asia

In Asia, the clinical use of genus* Phyllanthus* is very prevalent. The fruit of* P. emblica* has a long history of use in India and is called “amla” or “Indian gooseberry.” As a tonic in Indian Ayurveda, it is often used for liver diseases [3, 4]. This fruit is known as “yuganzi” in China. It has sweet and slightly astringent taste and is used for clearing heat from throat and moistening lung for arresting cough in Traditional Chinese Medicine (TCM). In Tibetan medicine this herb is used to treat blood and bile disease, and its preparations are clinically applicable to hypertension and anuria [2]. In Thailand, it is named “makham pom” and is employed to treat gastrointestinal chronic diseases.* P. emblica* is commonly used together with* Terminalia chebula* and* T. belerica* and called “Triphala.” “Triphala” is used as a clinical treatment protocol of gastropathy in India and as a remedy for pestilence and fatigue in China [62].

In India, fifteen species of genus* Phyllanthus* are widely used by indigenous medicine. These plants have bitter and astringent taste and are considered as stomachic, diuretic, febrifuge, deobstruent, antiseptic, and effective remedies for hepatopathy. Some herbs such as* P. niruri*,* P. amarus*,* P. fraternus*,* P. debilis*, and* P. maderaspatensis* share the same name “bhuiamlki” [29]. The fruits of “bhuiamlki” are employed by Ayurveda to cure jaundice.* P. simplex*,* P. reticulatus*, and* P. acidus* are therapy of urinary disease and have the names of “bhuiaveli,” “pancoli,” and “harfarauri,” respectively. The leaves of* P. polyphyllus*, called “sirunelli,” are used for liver disease. Additionally, the rest of these herbs can be employed as remedies for diabetes, jaundice, wound, fever, and inflammation.

In China, five herbs are commonly used by TCM, Tibetan medicine, Dai People, and Yi People [2]. They have bitter and sweet taste and are usually used as prescriptions. The whole plant of* P. urinaria*, known as “yexiazhu,” can clear heat-toxin and remove dampness and is employed to treat jaundice, enteritis, diarrhea, and dropsy. Besides, the TCM prescription, named “yexiazhu capsule,” performs a beneficial role in curing hepatitis B. Other herbs such as* P. reticulatus*,* P. niruri*, and* P. simplex* are beneficial to the treatment of ophthalmopathy, urinary infection, inflammation, and rheumatism.

In Thailand, eight herbs of this genus are widely used by residents.* P. amarus*,* P. urinaria*, and* P. virgatus* share the name “look tai bai,” all of which are used for treating gonorrhea, jaundice, diabetic, and liver disease.* P. acidus* has three names: “otaheiti gooseberry,” “star gooseberry,” and “mayom,” and it can be used as remedy for hypertensive, constipation, skin disease, and fever. The rest of herbal drugs including* P. taxodiifolius*,* P. niruri*, and* P. reticulatus* are employed for the treatment of urination disorder and malaria.

### 2.2. Africa

Many African tribes employ six plants of genus* Phyllanthus* to treat malaria, fever, and wound.* P. muellerianus* is the most popular herbal drugs of this genus in Africa. It is named “mbolongo” in Cameroon. In Ghana and Cameroon, the stem bark is used for the therapy of wound and tetanus. In Nigeria, Zambia, and Ivory Coast the leaves and root are applied as a fever remedy. In Kenya, the root of* P. polyanthus* is used to cure sexually transmitted diseases. What is more, the whole plants of* P. muellerianus* and* P. reticulatus* can be used for the treatment of malaria.

### 2.3. Latin America

About six herb species of this genus are used in many countries in Latin America. In Brazil,* P. tenellus* is popularly known as “quebra-pedras” whose leaves can be used as diuretic.* P. amarus* is named “chanca piedra” in Peru and the leaves are employed for diabetic and jaundice therapy or as sedative and astringent.* P. sellowianus* is called “sarandi blanco” in South America and used widely in folk for the treatment of urination disorder and diabetes.

In summary,* P. emblica*,* P. reticulatus*, and* P. niruri* are the top three species widely used around the world.* P. niruri* is probably the most widespread herb of* Phyllanthus*, which is named “chanka piedra,” “bhuiamlki,” “zhuzicao,” “dukung anak,” “quebra-pedra,” and “chanca piedra.” Its whole plant can treat inflammation, lithiasis, fever, malaria, hepatitis, and gonorrhea [7, 18, 19, 21, 22].

## 3. Chemical Constituents

More than 510 compounds have been isolated from* Phyllanthus*, the majority of which are lignins, triterpenoids, flavonoids, and tannins. The compositions isolated from each species and their biological activities are partially summarized in Table 2. Lignins and tannins exhibit various activities and are considered to be the biological active compounds of this genus. Corilagin, geraniin, and gallic acid are three most prevalent compounds in this genus, and the pharmacological researches mainly focus on phyllanthin, niranthin, and geraniin.

### 3.1. Terpenoids

Terpenoids are the most prevalent chemical class of the genus. About 125 compounds including 69 triterpenoids (1–69), 40 sesquiterpenes (70–109), 11 diterpenoids (110–120), and 5 monoterpenes (121–125) are mainly identified from* P. flexuosus*,* P. reticulatus*,* P. watsonii*,* P. emblica*,* P. acuminatus*, and* P. veuminatus*. Compounds 1–14 are tetracyclic triterpenoids, and compounds 15–69 are pentacyclic triterpenoids. In pentacyclic triterpenoids, compounds 15–36, compounds 37–49, and compounds 50–65 are oleanane type, friedelane type, and lupine type, respectively. Glochidone and lupeol are representatives of lupine type triterpenoids, which were suggested to have antitumor activities and mainly isolated from* Phyllanthus* species [68, 80, 96].

### 3.2. Phenylpropanoids

Phenylpropanoids (126–227) have typical C6–C3 constituents, which chiefly involve three groups including lignins, simple phenylpropanoids, and coumarins. 90 lignins (126–215) have been isolated from genus* Phyllanthus* since 1944. Compounds 126–176 are arylnaphthalene type lignins with a ring caused by the link of C-6 and C-7′. Compounds 177–190 are dibenzylbutane type lignins with two simple phenylpropanoids bounded by C-8 and C-8′. Phyllanthin, which had been studied to the most extent, was considered to be correlated with anti-inflammatory, immunomodulatory, antitumor, and hypotensive activities [127, 144, 163]. Pharmacokinetic studies of retrojusticidin B, a potential anti-HIV compound, had been done. The oral bioavailabilities dissolved in Tween 80 and in corn oil were found to be 22.1 and 33.1%, respectively [152].

### 3.3. Tannins

Tannins were progressively reported from the genus* Phyllanthus* since 1992. Hydrolyzable tannins (228–270) are characterized by the presence of one or more galloyl, hexahydroxydiphenoyl (HHDP), and HHDP metabolites attached to a glucopyranose core, which are mainly isolated from* P. emblica*,* P. amarus*,* P. niruri*, and* P. urinaria*. Compounds 271–279 are condensed tannins, which are the condensation of flavan-3-ols and linked by C-C. A great many condensed tannins were proved to have antiviral activity [53]. Ellagitannins (232–270) are the largest group of hydrolyzable tannins. Corilagin and geraniin are most extensively obtained from this genus and are characteristic compounds of ellagitannins, which exhibited multiple activities such as antioxidant, anti-HIV, antitumor, and antihyperalgesic activities [6, 111, 188, 195, 196, 199, 201, 202].

### 3.4. Flavonoids

Compounds 281–334 are flavonoids, which mainly contain flavonols (280–309), flavones (310–317), flavonones (318–324), flavan-3-ols (325–330), flavanonols (331), and isoflavone (332-333). Flavan-3-ols are the basic constitution of condensed tannins. Flavonols such as quercetin, quercitrin, and rutin demonstrated anti-inflammatory and antioxidant activities [151, 171, 178, 195, 203, 224].

### 3.5. Sterols

Until now, thirty sterols (334–363) from* Phyllanthus* have been reported. All the sterols are phytosterols with a side chain (C8–C10) substitution at C-17, and half of which were isolated from* P. emblica*.

### 3.6. Alkaloids

Thirty-two alkaloids (364–395) have been found in genus* Phyllanthus*, most of which are securinine and securinine-related compounds and mainly distributed in* P. niruri*. Compounds 390-391 isolated from* P. fraternus* are amide type alkaloids and exhibited antimalarial potential [250].

### 3.7. Phenols and Others

Compounds 396–468 belong to phenols, which have one and several phenolic hydroxyl groups. Thirty other constitutions (469–512) have been isolated. Mucic acid (compounds 445–455) and its derivatives (compounds 498-499) can only be found in* P. emblica* among this genus.

## 4. Biological Activity

The remarkable traditional uses of genus* Phyllanthus* lead to the various researches of biological activities, such as antiviral, antioxidant, antidiabetic, anticancer, and immunomodulatory activities. In this section, biological activity researches of the extracts of the plants are highlighted.

### 4.1. Antiviral Activity

Various* Phyllanthus* plants were reported to have strong antiviral potential such as anti-HIV, anti-HCV, anti-HSV, and anti-HCMV. The aqueous extract of* P. emblica* reduced viral load of HIV significantly at the dose of 400 *μ*g/mL [282]. DNA-polymerase and ribonuclease H (RNase H) activities of HIV-1 reverse transcriptase were inhibited by aqueous extract of* P. sellowianus* with IC_50_ values of 2.4 ± 0.8 *μ*g/mL and 5.9 ± 1.4 *μ*g/mL, respectively [283]. Moreover, methanol extract of* P. reticulatus* strongly inhibited the activity of RNase H by 99% at the dose of 50 *μ*g/mL [284].

HCV-infected HuH7 cells were used to test the anti-HCV activities of methanolic fraction of* P. amarus*. The fraction was proved to suppress the replication of HCV monocistronic replicon RNA and HCV H77S viral RNA without toxic effect in host cells. Inhibiting HCV-NS3 protease enzyme and NS5B enzyme may be the main mechanism [285]. Aqueous extract of* P. orbicularis* revealed inhibition activity against the replication of HCMV, HSV-1, and HSV-2 as well as BHV-1 with EC_50_ values of 57.7, 28.8, 25.7, and 21.27 *μ*g/mL, respectively. The selectivity indexes (SI) were ranged from 8.7 to 37.6 [286, 287].

Friend murine leukemia virus (FMuLv) induced erythroleukemia in BALB/c mice was relieved by metabolic extract of* P. amarus*. The extract inhibited leukemic cells from infiltrating into the sinusoidal space, decreased the morbidity of anemia, and improved survival rate of leukemia animals. Besides, the extract induced the upregulation of p53 and p45NFE2 and downregulation of Bcl-2 in the spleen [288].

### 4.2. Antioxidant Activity

Methanolic and aqueous parts of this genus have remarkable antioxidant activity, which may be correlated with the hydroxyl rich compositions.* P. acidus*,* P. polyphyllus*, and* P. fraternus* showed remarkable hepatoprotective activity against liver toxicity which was induced by acetaminophen, carbon tetrachloride, bromobenzene, and thioacetamide [42, 289–291]. The biochemical parameters as well as antioxidants levels were restored by these parts at the dose of 300 mg/kg. What is more, mitochondrial dysfunction in liver, induced by bromobenzene, was relieved by prior oral administration of aqueous part of* P. fraternus* at the dose of 100 mg/kg [51, 291].

Antimycin A governed mitochondrial protein degeneration, lipid peroxidation and mitochondrial DNA damage, and H_2_O_2_ induced membrane damage of Hep3B cells were considerably mitigated by aqueous fraction of* P. amarus* [164]. Mutagenesis induced by PhIP and 4-ABP and DNA damage induced by *γ*-ray and UVB were protected by aqueous fraction of* P. orbicularis* [292–294].

Methanol extract of* P. debilis* showed strong antioxidant activity when tested by various antioxidant assays including total antioxidant, free radical scavenging, superoxide anion radical scavenging, hydrogen peroxide scavenging, and nitric oxide scavenging assays. Besides, further study demonstrated that total phenolic was correlated with antioxidant activity [52]. In addition, hydromethanolic extract of* P. virgatus* exhibited substantially antioxidant capacity in both DPPH scavenging (IC_50_ = 30.4 *μ*g/mL) and linoleic acid oxidation inhibiting (84%) method [5].

### 4.3. Antidiabetic Activity

Twelve herb drugs such as* P. emblica*,* P. reticulatus*,* P. niruri*,* P. amarus*,* P. urinaria*,* P. acidus*,* P. debilis*,* P. virgatus*,* P. sellowianus*,* P. rheedii*,* P. orbicularis*, and* P. hookeri* are traditionally employed for diabetes in many countries. Recent researches about the hypoglycemic effect of* Phyllanthus* plants were abundant. Streptozotocin- and alloxan-induced diabetic rats were employed for the evaluation of antidiabetic potential of* P. emblica*,* P. niruri*,* P. reticulatus*,* P. sellowianus*,* P. virgatus*, and* P. simplex* [4, 295–299]. After oral administration of these (aqueous, methanol, and ethanol) extracts for 21–45 days, the concentration of blood glucose was significantly reduced, and the effects of* P. sellowianus* and* P. simplex* were similar to the glibenclamide group (10 mg/kg). In addition, methanol fraction of* P. virgatus* considerably inhibited the activity of *α*-amylase in the noncompetitive pattern with IC_50_ of 33.20 ± 0.556 *μ*g/mL [300].

After oral aqueous extract of* P. niruri* for 28 days, the levels of LPO and MDA were decreased while the concentrations of SOD, CAT, and GPx were increased. After being pretreated with the aqueous fraction of* P. sellowianus*, hemorheological parameters were ameliorated and red blood cells (RBCs) showed large globular aggregates and agglutination [301].

### 4.4. Anticancer Activity

Different extracts of the plants have been assessed for anticancer effects and the related mechanisms. Cancer cell lines such as NCI-H1703, MDA-MB-231, HeLa, 143B, PC-3, MCF-7, HepG2, A549, SKOV3, and HT-29 were considerably inhibited by* P. emblica*,* P. urinaria*,* P. polyphyllus*,* P. watsonii*, and* P. pulcher* [57, 68, 302–309]. In addition,* P. emblica* showed no toxicity to normal cells (MRC5). The extracts inhibited growth of cells through fragmentation of DNA and dysfunction of mitochondrial including upregulated mitochondrial fission 1 protein and downregulated optic atrophy type 1 and mitofusin 1 [304]. Moreover, the extracts suppressed the ability of cell invasion, migration, and adhesion. Further researches demonstrated that the fractions induced apoptosis, invasion, and migration through increasing the expression of caspase-3, caspase-7, caspase-8, and p-JNK and decreasing the expression of ERK, p-ERK1/2, JNK, MMP-2, MMP-9, Wnt, NF-*κ*B, Myc/Max, and hypoxia [302, 303, 307].

Ehrlich ascites carcinoma tumor model was used to evaluate the antitumor activity of* P. polyphyllus*. Oral administration of methanol fraction at the dose of 200 mg/kg could significantly reduce the solid tumor volume. Hematological parameters, protein, packed cellular volume (PCV), and antioxidant enzymes such as LPO, GPx, GST, SOD, and CAT were greatly regulated [57].

### 4.5. Immunomodulatory Activity

Ethanol extracts of* P. urinaria* and* P. amarus* were demonstrated to have inhibitory effects on the chemotaxis of neutrophils and monocytes with IC_50_ lower than 2.92 *μ*g/mL. In addition, phagocytic activity and CD18 expression of neutrophils and monocytes were downregulated [163].

Oral administration of* P. reticulatus* extract at the dose of 100 mg/kg demonstrated a significant increase in phagocytic activity, the percentage of neutrophil adhesion, and white blood cell in albino mice [310].

### 4.6. Analgesic Activity

The extracts of* P. corcovadensis*,* P. niruri*, and* P. tenellus* showed significant reduction in writhing response induced by acetic acid, with ID_50_ values of 30, 19, and >30 mg/kg, respectively. The late phase of formalin-induced pain could be relieved by* P. tenellus* with ID_50_ of 100 mg/kg and both phases of formalin-induced pain could be reduced by* P. corcovadensis* and* P. niruri* with ID_50_ values of 100 and 52 mg/kg, respectively. The analgesic effects could not be antagonized by naloxone [311]. In addition, intraperitoneally given hydroalcoholic extracts of* P. amarus*,* P. orbicularis*, and* P. fraternus* produced a marked analgesic activity by inhibiting acetic acid-induced abdominal constriction, capsaicin-induced neurogenic pain, and late phase of formalin-induced paw licking [312]. The ethanol and aqueous extracts of* P. emblica* succeeded in inhibiting acetic acid-induced writhing response but failed in the tail-immersion test [313].

### 4.7. Anti-Inflammatory Activity

In recent years, different inflammatory models such as Freund's complete adjuvant induced arthritis, carrageenin induced paw edema, and cotton pellet induced granuloma were employed to evaluate the anti-inflammatory effect of* Phyllanthus*. After receiving the aqueous extract of* P. amarus*, indexes of arthritis, joint diameter, and paw volume were decreased and thresholds of mechanical hyperalgesia and nociceptive were increased [314]. The ethanol fraction of* P. simplex* ameliorated the parameters of paw edema and granuloma and substantially inhibited nitric oxide (NO) production [315].

### 4.8. Antispasmodic Activity

Isolated rabbit jejunum and guinea-pig ileum were employed for the* in vitro* tests for the antispasmodic effects of* P. emblica*. Carbachol and K^+^ induced contractions of rabbit jejunum were released by the extract with IC_50_ values of 0.09 mg/mL and 1.38 mg/mL. The pretreatment of guinea-pig ileum with the extract at 0.3 mg/mL caused a rightward parallel shift in the concentration-response curves of acetylcholine without suppression of the maximum contractile response. Dual blockade of muscarinic receptors and Ca^2+^ channels can explain its antispasmodic activity [316].

### 4.9. Hypotensive and Hypolipidemic Activity

Aqueous extract of the leaves of* P. amarus* was found to restrain both force and rate of myocardial contraction and to inhibit the intrinsic myogenic contraction of isolated rat portal vein [317]. Aqueous part of* P. reticulatus* was effective in releasing total cholesterol, lipid profile, and oxidative stress in hypercholesterolemic albino rats after oral administrated for 45 days at 250 mg/kg [14].

### 4.10. Wound Healing

Extracts of* P. emblica* and* P. niruri* were demonstrated to have wound healing effect. Topical application with* P. emblica* could promote the proliferation of cells and cross-link of collagen in the full thickness excision wound [318]. Oral administration of* P. emblica* at the dose of 60 mg/kg showed healing effect against NSAID-induced gastric ulcer through upregulating the concentration of IL-10 and downregulating the levels of TNF-*α* and IL-1*β* [319]. After treatment with* P. niruri* at the dose of 200 mg/kg, 98.8% of wound could be recovered in the excision and incision wound models on the 16th day [320].

### 4.11. Antimalarial Activity

Malaria is a prevalent disease in many tropical and subtropical countries and folks of these places especially African people employed* Phyllanthus* as antimalarial agency.* Plasmodium falciparum* was suppressed by ethyl acetate fraction of* P. acidus* with IC_50_ of 9.37 *μ*g/mL, and the SI equals 4.88 for HEp-2 cells and 11.75 for Vero cells [321]. What is more, chloroquine-resistant* P. falciparum* could be exhibited by* P. amarus* and* P. muellerianus* with IC_50_ values of 11.7 and 9.4 *μ*g/mL, respectively.* P. amarus* presented protection effect on human RBCs damage caused by the virus [322]. The SI of* P. muellerianus* was higher than 5.3 for L-6 and MRC-5 cell lines [25, 202].

### 4.12. Antidepressant Activity

The aqueous extract of* P. emblica* (200 mg/kg) significantly decreased immobility period in both tail suspension test and forced swim test by decreasing the levels of MAO-A and GABA [323]. In the plus-maze, Hebb-Williams maze, and passive avoidance apparatus test, preparation of* P. emblica* produced a dose-dependent upgrade in scores. The preparation was also proved to reverse the amnesia induced by diazepam and scopolamine and to reduce the cholinesterase activity and total cholesterol level in brain [324, 325].

### 4.13. Others

The essential oil fraction of* P. muellerianus* exhibited strong antibacterial activity against* Clostridium sporogenes*,* Streptococcus mutans*, and* S. pyogenes* with MIC values ranging from 13.5 to 126 *μ*g/mL [326]. Methanol extract of* P. acuminatus* (100 mg/mL) showed stronger antifungal than Dithane M-45 (10 000-ppm solution) against* Pythium ultimum* [327].

Aqueous extract of* P. acidus* was proved to regulate electrolyte transport in cystic fibrosis airways by increasing the intracellular levels of cAMP and Ca^2+^, stimulating basolateral K^+^ channels, and activating and redistributing cellular localization of cystic fibrosis transmembrane conductance regulator [328].

Eight hours after being treated with the aqueous extract of* P. sellowianus* at a dose of 400 mg/kg, urine output of test animals was decreased from 2.59 to 3.69 mL/100 g [329].

## 5. Clinical Studies

The extracts of* P. niruri* were proved to have immunomodulatory effect and played a crucial role in treating pulmonary tuberculosis and vaginal candidiasis as well as varicella. In patients with pulmonary tuberculosis, after oral administration of* P. niruri* 50 mg/mL for 2–6 months, the level of IL-10 was decreased and the levels of plasma IFN-*γ* and TNF-*α* were significantly increased. After 1-month treatment, the increase of the ratio of CD4^+^/CD8^+^ was observed. In the vaginal candidiasis patients, after receiving* P. niruri* 100 mg/mL for 1–3 months, the levels of IFN-*γ* and IL-12 were elevated. As for varicella patients, the number of papules and the number crusts were decreased after treatment with the extract at the dose of 5 mg/mL [330].

Clinical studies of* P. niruri* in Brazil had been finished, from which the* P. niruri* showed beneficial effects on the treatment of urolithiasis. After 3-month treatment, calculi elimination was increased. Furthermore, urinary calcium excretion and residual stone fragments after lithotripsy were decreased. Toxic effects on kidney, cardiovascular, and nervous systems were not found [331].

In China, the clinical study of* P. urinaria* in treating chronic hepatitis B with 140 patients was well established. The results indicated that, after treatment with* P. urinaria* capsule for 3 months or 2 years, especially in the long term, the recovery rate in the index of HBV-DNA and HBeAg was 88.2% and 52.5%, respectively. Once the treatment stopped, the recurrence rate was 10.4% to 13.4% [332].

## 6. Toxicity Studies

After given aqueous leaf extract of* P. niruri* at the dose of 2000 mg/mL, no acute toxicity was observed at the levels of bilirubin, ALT, AST, total protein, albumin, globulin, ALP, GGT, urea, creatinine, full blood count, and hemoglobin [333]. After being treated with ethanol extract of* P. niruri* over a period of 90 days at doses of 30 and 300 mg/kg, the rats showed no genotoxic effect at the test of PCE/NCE ratio [334]. Reproductive toxicity of* P. niruri* was tested using estrogen values, progesterone values, and testosterone levels. The estrogen and progesterone levels increased more than 1.5-fold above the control group after receiving 50 and 500 mg/kg aqueous leaf extract for 90 days, which reminded us of the cytotoxic of male antifertility properties [335].

Nephrotoxicity including interstitial oedema and tubular necrosis were detected after receiving 400 and 800 mg/kg of aqueous extract from* P. amarus* for 30 days [336]. The test animals were given 800 and 1600 mg/kg of the aqueous extract of* P. amarus* for 10 days, and significant pathological changes were found in the liver, kidney, and testis. The frequency of MNPCE, sperm abnormalities, total white blood cell, and lymphocyte counts were significantly increased, which suggested the genetic and systemic toxicity of* P. amarus* [337]. In addition, aqueous, methanolic, and hydromethanolic extracts of* P. amarus* (400 mg/kg) reduced locomotor activity and showed CNS depressant effect [338].

The LD_50_ of ethanolic extract from* P. fraternus* was 1125 mg/kg in the toxicity test. When the rats received the extract at doses of 400 mg/kg for 7 days, no toxicity was detected in liver and kidney [339]. Hydroethanolic extract* P. fraternus* showed the quick onset and long duration of reduction of locomotor activity at the dose of 400 mg/kg [338].

## 7. Conclusion

514 compounds have been isolated from different species of* Phyllanthus*, including 126 terpenoids, 102 phenylpropanoids, 73 phenols, 54 flavonoids, 53 tannins, 33 sterols, 31 alkaloids, and a number of other compositions. Their wide range of biological activities such as antiviral, antioxidant, antidiabetic, anticancer, anti-inflammatory, hypolipidemic, immunomodulatory, and antidepressant activities are tested using polar solvents (water, methanol, and ethanol) extracts. These extracts are considered rich in phenols, flavonoids, and tannins, which may exhibit antioxidant activity in different degree due to their hydroxyl [340]. Consequently, most bioactivities of* Phyllanthus* may be correlated with the hydroxyl rich compounds.

In recent years, the traditional uses of* Phyllanthus* had been partly confirmed, and more evidences such as pharmacological researches and clinical studies are urgently needed to be taken. Further studies of phytochemical discovery and subsequent screenings are necessary to be taken to extend the use of* Phyllanthus* and to develop leading compound.

## Figures and Tables

**Table 1:** The traditional use of *Phyllanthus*.

Species	Region	Local name	Plant part used	Traditional use	Reference
*P. emblica*	Bangladesh		Fruit	Constipation, urinary diseases	[8]
	Burma		Juice/bark	Constipation, hemostasis, keratitis	[8]
	Cambodia		Leaves	Muscle pain, fever	[8]
	China	Yuganzi	Fruit	Digestive disease, hypertension, fever, respiratory inflammation	[8]
	Fiji		Fruit	Tonic	[8]
	India	Amla, Indian gooseberry	Fruit	Diabetes, chronic diarrhea, inflammation, fever, liver diseases, stomach ulcers, metabolic disorders, skin disorders, beauty care	[3, 4]
	Indonesia		Leaves/fruit	Diarrhea, abdominal pain, stomach Disease, gallbladder disease, bleeding	[8]
	Iran		Fruit	Parasitic	[8]
	Iraq		Fruit	Bleeding, gastrointestinal system disorder	[8]
	Nepal		Stem/fruit/seed	Urination disorder, constipation, bleeding, diarrhea, ophthalmopathy, asthma, bronchitis	[8]
	Pakistan		Fruit	Diarrhea, preterm, skin diseases, gonorrhea, ophthalmopathy, anemia, hair care	[8, 9]
	Sri Lanka		Fruit/whole plant	Constipation, indigestion, keratitis	[8]
	Thailand	Makham pom	Juice/bark	Diarrhea, leukorrhagia, cough, parasitosis, gastrointestinal chronic diseases, hair treatment and nourishment, skin care	[8, 10, 11]
	Turkey		Fruit	Diarrhea, dysentery, hemostasis, gastroenteritis	[8]

*P. reticulatus*	Bangladesh		Whole plant	Edema, constipation, helminthiasis, dysentery, diarrhea, pain	[12]
	China	Huangguo yexiazhu		Inflammation, rheumatism	[13]
	India	Pancoli, karineli	Leaves/bark	Urination disorder, fever, smallpox, colic, constipation, diabetes	[12, 14, 15]
	Kenya			Malaria	[7]
	Malaysia		Leaves	Smallpox, syphilis, asthma, diarrhea, bleeding from gums, diabetes, urination disorder, sores, burn, suppuration, chafe, venereal sores	[16, 17]
	Sri Lanka		Bark/fruit	Enteritis, urination disorder	[15]
	Sudan			Urination disorder, fever	[15]
	Tanzania		Whole plant/leaves	Dysmenorrhea, gonorrhea, urination disorder, intestinal hemorrhage and anemia, muscle spasms, diarrhea with anal bleeding, promoting fertility, sores	[12, 15]
	Thailand			Urination disorder, asthma, anemia, fever, thirst, astringent, inflammation	[16]

*P. niruri*	Brazil	Quebra-pedra	Whole plant	Kidney calculi	[18]
	China	zhuzicao	Whole plant	Hepatitis, dysentery, enteritis, urinary infection	[19]
	Congo		Whole plant	Malaria	[20]
	India	Chanka piedra, bhuiamlki	Fruit/whole plant	Bronchitis, anaemia, leprosy, asthma, kidney calculi, ulcer, wound, sore, scabies, ring worm, jaundice, gonorrhea, menstruation, diabetes	[18, 21–23]
	Indonesia		Whole plant	Viral infection, hepatitis	[22]
	Latin America	Chanca piedra	Whole plant	Gallstone, kidney calculi, fever, excess uric acid	[6, 18, 24]
	Malaysia	Dukong anak	Whole plant	Diarrhoea, kidney disorder, gonorrhea, cough	[22]
	Thailand		Aerial parts	Anorexia, malaria	[18]

*P. muellerianus*	Africa			Malaria	[25]
	Cameroon	Mbolongo	Stem bark	Wound, tetanus	[26]
	Ghana			Wound	[27]
	Ivory Coast		Leaves	Fever	[26]
	Nigeria		Root	Fever	[26]
	Zambia		leaves	Fever	[26]

*P. amarus*	Africa		Whole plant	Urinary concretions, dysentery, jaundice, diarrhoea	[28]
	India	Bhuiamlki	Whole plant	Gastropathy, diarrhoea, dysentery, intermittent fevers, ophthalmopathy, scabies, ulcers, wound, malaria, jaundice, diabetes, asthma, hepatitis, tuberculosis, urinary diseases, bodyache, immunomodulatory	[29–34]
	Nigeria		Leaves	Diabetes mellitus, obesity, hyperlipidemia, malaria	[35, 36]
	Peru	Chanca piedra	Leaves	Diabetes, jaundice, kidney diseases, urination disorder, sedative, astringent, tonic	[37]
	Thailand	Look tai bai		Gonorrhea, jaundice, diabetes, liver diseases	[5]

*P. urinaria*	China	Yexiazhu	Whole plant	Kidney calculi, painful disorder, jaundice, enteritis, diarrhea, dropsy, inflammation	[38–41]
	India			Inflammation, diarrheal, kidney calculi, painful disorder	[38, 39]
	Thailand	Look tai bai		Inflammation, diarrheal, gonorrhea, jaundice, diabetes	[5, 38]

*P. acidus*	India	Harfarauri	Fruit/leaves/roots	Jaundice, constipation, vomiting, biliousness, urinary concretions, piles, fever, smallpox, rheumatism, asthma, hepatic disease, diabetes, gonorrhea, ophthalmopathy, amnesia, psoriasis	[42, 43]
	Thailand	Otaheiti gooseberry, star gooseberry, mayom	Leaves/bark/root	Constipation, alcoholic addicts, hypertension, fever, dermatitis, menstruation fever	[44–46]

*P. debilis*	India	Bhuiamlki		Swelling, intestinal worms, fever, wound, inflammation, rheumatism	[34]
	Sri Lanka			Diabetes	[47]

*P. simplex*	India	Bhuiaveli, uchchiyusirika	Leaves/whole plant	Ophthalmopathy, gonorrhea, jaundice, mammary abscess, pruritus, diarrhea, hepatitis, urinary infection	[48, 49]
	China	Huang zhuzicao		Ophthalmopathy, diarrhea, hepatitis, urinary infection	[49]

*P. discoideus *	Cameroon			Insomnia, epilepsy	[50]

*P. fraternus*	India	Bhuiamlki	Whole plant	Constipation, jaundice, hepatic disorder, kidney disorders, bacterial infection	[29, 51, 52]

*P. hookeri*	India			Diabetes, wound, fever, inflammation, snake bite, bacterial infection	[34]

*P. kozhikodianus*	India			Dysentery, jaundice, ulcer, itching, bacterial infection	[34]

*P. maderaspatensis*	India	Bhuiamlki	Whole plant	Headache, constipation, diarrhea, edematous, dysentery, fever, ulcer, burn, jaundice, bacterial infection, immunomodulatory	[34, 52]

*P. nozeranii *	India			Spasmodic, piles, headache, boils, indigestion, viral and bacterial infection	[34]

*P. orbicularis*	Cuba			Jaundice, diabetes, kidney calculi, ulcer, rheumatism, fever	[53, 54]

*P. piscatorum*	Venezuela		Aerial parts	Wound, fungal infection	[55]

*P. polyanthus*	Kenya		Root	Sexually transmitted diseases	[56]

*P. polyphyllus*	India	Sirunelli	Leaves	Liver disease	[57]

*P. rheedii *	India		Whole plant	Diabetes	[58]

*P. sellowianus *	South America	Sarandi blanco	Stems/leaves	Urination disorder, diabetes	[59]

*P. taxodiifolius*	Thailand		Leaves/twigs	Urination disorder	[60]

*P. tenellus*	Brazil	Erva pombinha, quebra-pedra	Leaves	Urination disorder, kidney calculi	[61]

*P. virgatus*	Thailand	Look tai bai		Gonorrhea, jaundice, diabetes, liver disease	[5]

**Table 2:** The compounds isolated from the genus *Phyllanthus* and part of pharmacological effects.

Number	Compounds	Species	Pharmacological effects	References
1	(20S)-3*α*-Acetoxy-24-methylenedammaran-20-ol	*P. polyanthus *		[56]
2	(20S)-3*β*-Acetoxy-24-methylenedammaran-20-ol	*P. polyanthus *		[56]
3	Ocotillol-II	*P. flexuosus*		[63]
4	Phyllanthenol	*P. niruri*		[64]
5	Phyllanthenone	*P. niruri*		[64]
6	Phyllantheol	*P. niruri*		[64]
7	(+)-Songbodichapetalin	*P. songboiensis*		[65]
8	Acutissimatriterpene A	*P. acutissima*		[66]
9	Acutissimatriterpene B	*P. acutissima*		[66]
10	Acutissimatriterpene C	*P. acutissima*		[66]
11	Acutissimatriterpene D	*P. acutissima*		[66]
12	Acutissimatriterpene E	*P. acutissima*		[66]
13	Flexuosoids A	*P. flexuosus*		[67]
14	Flexuosoids B	*P. flexuosus*		[67]
15	*δ*-Amyrin acetate	*P. polyanthus *		[56]
16	12(13)-Dehydro-3*α*-acetoxyolean-28-oic acid	*P. pulcher*		[68]
17	3′-O-Acetyl-3-O-*α*-L-arabinosyl-23-hydroxyolean-12-en-28-oic acid	*P. polyphyllus*		[69]
18	3*α*-Acetoxyl-25-hydroxyolean-12-en-28-oic acid	*P. pulcher*	Antitumor	[68]
19	4′-O-Acetyl-3-O-*α*-L-arabinosyl-23-hydroxyolean-12-en-28-oic acid	*P. polyphyllus*		[69]
20	Olean-12-en-3*β*,15*α*,24-triol	*P. flexuosus*	Antitumor	[70, 71]
21	Olean-12-en-3*β*,15*α*-diol	*P. flexuosus*	Antitumor	[70, 71]
22	Olean-12-en-3*β*,24-diol	*P. flexuosus*		[70]
23	Olean-18-en-3*α*-ol	*P. fraternus*		[72]
24	Oleana-11:13(18)-dien-3*β*-ol	*P. flexuosus*		[70]
25	Oleana-11:13(18)-dien-3*β*,24-diol	*P. flexuosus*		[70]
26	Oleana-9(11):12-dien-3*β*-ol	*P. flexuosus*		[70]
27	Oleanolic acid	*P. urinaria*		[73]
28	Phyllanoside	*P. amarus*		[74]
29	Phyllenolide A	*P. myrtifolius*		[75]
30	Phyllenolide B	*P. myrtifolius*		[75]
31	Phyllenolide C	*P. myrtifolius*		[75]
32	Taraxerol	*P. columnaris*		[76]
33	Taraxerone	*P. reticulatus*		[77]
33	Taraxerone	*P. columnaris*		[76]
34	Taraxeryl acetate	*P. reticulatus*		[77]
35	*α*-Amyrin	*P. singampattiana*		[78]
36	*β*-Amyrin	*P. urinaria*		[79]
36	*β*-Amyrin	*P. flexuosus*		[80]
36	*β*-Amyrin	*P. acidus*		[81]
37	11*β*-Hydroxy-D:A-friedoolean-1-en-3-one	*P. flexuosus*		[82]
38	1*β*,22*β*-Dihydroxyfriedelin	*P. muellerianus*		[83]
39	21*α*-Hydroxyfriedel-4(23)-en-3-one	*P. reticulatus*		[84]
40	21*α*-Hydroxyfriedelan-3-one	*P. reticulatus*		[84]
41	22*β*-Hydroxyfriedel-1-ene	*P. muellerianus *		[83]
42	26-Nor-D:A-friedoolean-14-en-3-one	*P. watsonii*		[85]
43	26-Nor-D:A-friedoolean-14-en-3*β*-ol	*P. watsonii*		[85]
43	Friedelin	*P. columnaris*		[86]
44	3,20-Dioxo-dinorfriedelane	*P. emblica*		[87]
45	Epifriedelinol	*P. reticulatus*		[77]
45	Epifriedelinol	*P. singampattiana*		[78]
46	Friedelan-3*β*-ol	*P. reticulatus*		[84]
47	Friedelin	*P. niruri*		[88]
47	Friedelin	*P. reticulatus*		[84]
47	Friedelin	*P. flexuosus*		[80]
47	Friedelin	*P. watsonii*		[85]
47	Friedelin	*P. wightianus*		[89]
47	Friedelin	*P. singampattiana*		[78]
48	Polpunonic acid	*P. oxyphyllus*		[90]
49	Trichadenic acid B	*P. flexuosus*		[91]
50	3-Friedelanone	*P. muellerianus*		[92]
51	Betulin	*P. reticulatus*		[77]
51	Betulin	*P. flexuosus*	Antitumor	[70, 71]
52	Betulinic acid	*P. reticulatus*		[84]
53	Glochidiol	*P. urinaria*		[73]
53	Glochidiol	*P. sellowianus*		[93]
54	Glochidone	*P. virgatus*		[94]
54	Glochidone	*P. sellowianus*		[95]
54	Glochidone	*P. watsonii*		[85]
54	Glochidone	*P. taxodiifolius*	Antitumor	[60, 96]
54	Glochidone	*P. pulcher*	Antitumor	[68]
54	Glochidone	*P. flexuosus*		[80]
55	Glochidonol	*P. reticulatus*		[84]
55	Glochidonol	*P. sellowianus*		[93]
55	Glochidonol	*P. watsonii*		[85]
55	Glochidonol	*P. pulcher*	Antitumor	[68]
56	Lup-20(29)-en-3*β*,15*α*-diol	*P. flexuosus*	Antitumor	[63, 71]
57	Lup-20(29)-en-3*β*,24-diol	*P. flexuosus*	Antitumor	[70, 71]
58	Lup-20(29)-en-3*β*-ol	*P. urinaria*		[97]
59	Lup-20(29)-ene-3*β*,24-diol	*P. flexuosus*		[98]
60	Lup-20(29)-ene-1*β*,3*β*-diol	*P. sellowianus*		[93]
60	Lup-20(29)-ene-1*β*,3*β*-diol	*P. watsonii*		[85]
61	Lupanyl acetate	*P. urinaria*		[99]
61	Lupanyl acetate	*P. watsonii*		[85]
61	Lupanyl acetate	*P. columnaris*		[86]
61	Lupanyl acetate	*P. pulcher*		[68]
62	Lupenone	*P. polyanthus *		[56]
63	Lupenyl palmitate	*P. watsonii*		[85]
64	Lupeol	*P. emblica*		[100]
64	Lupeol	*P. urinaria*		[79]
64	Lupeol	*P. reticulatus*		[17]
64	Lupeol	*P. flexuosus*	Antitumor	[71, 80]
64	Lupeol	*P. oxyphyllus*		[90]
64	Lupeol	*P. watsonii*		[85]
64	Lupeol	*P. taxodiifolius*	Antitumor	[60, 96]
64	Lupeol	*P. wightianus*		[89]
64	Lupeol	*P. columnaris*		[86]
65	Lupeol acetate	*P. reticulatus*		[17]
66	29-Nor-3,4-seco-friedelan-4(23),20(30)-dien-3-oic acid	*P. oxyphyllus*		[90]
67	3,7,11,15,19,23-Hexamethyl-2Z,6Z,10Z,14E,18E,22E-tetracosahexen-1-ol	*P. niruri*		[101]
68	Phyllanthol	*P. sellowianus*		[102]
68	Phyllanthol	*P. polyanthus *		[56]
68	Phyllanthol	*P. acidus*		[81]
69	Phyllanthone	*P. polyanthus *		[56]
70	4′-Hydroxyphyllaemblicin B	*P. emblica*		[103]
71	5-Hydroxy-6,9-epoxyguaiane	*P. oxyphyllus*		[90]
72	5-O-Acetyl-6,9-epoxyguaiane	*P. oxyphyllus*		[90]
73	Cloven-2*β*,9*α*-diol	*P. urinaria*		[73]
74	Descinnamoylphyllanthocindiol	*P. acuminatus*		[104]
75	Didesacetylphyllanthostatin 3	*P. acuminatus*		[104]
76	Dihydrophaseic acid-4′-O-*β*-D-glucopyranoside	*P. reticulatus*		[105]
77	Englerins A	*P. engleri*	Antitumor	[106]
78	Englerins B	*P. engleri*		[106]
79	Glochicoccin D	*P. emblica*		[107]
80	Jaslanceoside B	*P. cochinchinensis*		[108]
81	Jasminoside	*P. cochinchinensis*		[108]
82	Phyllaemblic acid	*P. emblica*		[109]
83	Phyllaemblic acid B	*P. emblica*		[110]
84	Phyllaemblic acid C	*P. emblica*		[110]
85	Phyllaemblicin A	*P. emblica*		[109]
86	Phyllaemblicin B	*P. emblica*	Antiviral and antitumor	[109, 111, 112]
87	Phyllaemblicin C	*P. emblica*	Antitumor and antiviral	[109, 111, 113]
88	Phyllaemblicin D	*P. emblica*		[110]
89	Phyllaemblicin E	*P. emblica*		[103]
90	Phyllaemblicin F	*P. emblica*		[103]
91	Phyllaemblicin G1	*P. emblica*		[107]
92	Phyllaemblicin G2	*P. emblica*		[107]
93	Phyllaemblicin G3	*P. emblica*		[107]
94	Phyllaemblicin G4	*P. emblica*		[107]
95	Phyllaemblicin G5	*P. emblica*		[107]
96	Phyllaemblicin G6	*P. emblica*	Antiviral	[107]
97	Phyllaemblicin G7	*P. emblica*		[107]
98	Phyllaemblicin G8	*P. emblica*		[107]
99	Phyllaemblinol	*P. emblica*		[114]
100	Phyllanthocin	*P. brasiliensis*		[115]
101	Phyllanthoside	*P. acuminatus*	Antitumor	[116]
101	Phyllanthoside	*P. veuminatus*	Antitumor	[117]
101	Phyllanthoside	*P. brasiliensis*	Antitumor	[115]
102	Phyllanthostatin 1	*P. acuminatus*	Antitumor	[116]
102	Phyllanthostatin 1	*P. veuminatus*	Antitumor	[117]
103	Phyllanthostatin 2	*P. acuminatus*	Antitumor	[117]
103	Phyllanthostatin 2	*P. veuminatus*	Antitumor	[117]
104	Phyllanthostatin 3	*P. acuminatus*	Antitumor	[117]
104	Phyllanthostatin 3	*P. veuminatus*	Antitumor	[117]
105	Phyllanthostatin 6	*P. acuminatus*	Antitumor	[104]
106	Phyllanthusol A	*P. acidus*	Antitumor	[46]
107	Phyllanthusol B	*P. acidus*	Antitumor	[46]
108	*β*-Caryophyllene	*P. emblica*		[113]
109	*β*-Bourbonene	*P. emblica*		[113]
110	19-Hydroxyspruceanol 19-O-*β*-D-glucopyranoside	*P. reticulatus*		[118]
111	Cleistanthol	*P. urinaria*		[73]
111	Cleistanthol	*P. reticulatus*		[13]
111	Cleistanthol	*P. flexuosus*	Antitumor	[119]
111	Cleistanthol	*P. oxyphyllus*		[90]
112	Ent-3*β*-Hydroxykaur-l6-ene	*P. flexuosus*		[80]
113	Orthosiphol G	*P. niruri*		[120]
114	Orthosiphol I	*P. niruri*		[120]
115	Phyllanflexoid A	*P. flexuosus*	Antitumor	[119]
116	Phyllanflexoid B	*P. flexuosus*	Antitumor	[119]
117	Phyllanflexoid C	*P. flexuosus*		[119]
118	Phyllanterpenyl ester	*P. fraternus*		[121]
119	Spruceanol	*P. urinaria*		[73]
119	Spruceanol	*P. reticulatus*		[13]
119	Spruceanol	*P. oxyphyllus*		[90]
119	Spruceanol	*P. songboiensis*		[65]
120	*trans*-Phytol	*P. niruri*		[122]
121	(3S,5R,6S,9R)-Megastigmane-3,9-diol 3-O-*α*-L-arabinofuranosyl-(1 → 6)-*β*-D-glucopyranoside	*P. reticulatus*		[13]
122	(6R)-Menthiafolic acid	*P. urinaria*		[73]
123	7-Megastigmen-3-ol-9-one 3-O-*α*-L-arabinofuranosyl-(1 → 6)-*β*-D-glucopyranoside	*P. reticulatus*		[13]
124	Turpenionoside A	*P. reticulatus*		[118]
125	Turpenionoside B	*P. reticulatus*		[118]
126	7-O-[(2,3,4-Tri-O-acetyl)-*α*-L-arabinopyranosyl]diphyllin	*P. poilanei*	Antitumor	[123]
127	Arabelline	*P. flexuosus*		[67]
128	Acutissimalignans A	*P. songboiensis*		[65]
128	Acutissimalignans A	*P. acutissima*		[66]
129	Cleistanthin A	*P. taxodiifolius*	Antitumor	[96, 124]
130	Cleistanthin A acetate	*P. taxodiifolius*	Antitumor	[96, 124]
131	Cleistanthin A Me ether	*P. taxodiifolius*	Antitumor	[96, 124]
132	Cleistanthin B	*P. poilanei*		[123]
133	Cleistanthoside A	*P. taxodiifolius*		[96]
134	Cleistanthoside A tetraacetate	*P. taxodiifolius*	Antitumor	[96, 124]
135	Dextrobursehernin	*P. urinaria*		[125]
136	Diphyllin	*P. poilanei*		[123]
136	Diphyllin	*P. polyphyllus*	Anti-inflammatory	[126]
137	Hypophyllanthin	*P. niruri*	Hepatoprotection and hypotensive	[127–129]
137	Hypophyllanthin	*P. urinaria*	Hypotensive	[125, 130]
137	Hypophyllanthin	*P. virgatus*		[131]
137	Hypophyllanthin	*P. amarus*	Antitumor and anti-CYP3A4	[132–134]
137	Hypophyllanthin	*P. debilis*		[135]
138	Isolariciresinol	*P. emblica*		[114]
139	Isolintetralin	*P. niruri*		[136]
139	Isolintetralin	*P. urinaria*		[125]
139	Isolintetralin	*P. virgatus*		[131]
140	Justicidin A	*P. myrtifolius*		[131]
141	Iusticidin B	*P. myrtifolius*		[137]
141	Iusticidin B	*P. polyphyllus*	Anti-inflammatory	[126]
141	Iusticidin B	*P. anisolobus*		[138]
141	Iusticidin B	*P. piscatorum*	Antifungal, antitumor, and antiparasitic	[139]
142	Lintetralin	*P. niruri*		[128]
142	Lintetralin	*P. urinaria*		[125]
143	(+)-Lyoniresinol	*P. reticulatus*		[13]
144	(+)-Lyoniresiol	*P. urinaria*		[73]
145	Mananthoside I	*P. reticulatus*		[118]
146	Neonirtetralin	*P. niruri*		[140]
146	Neonirtetralin	*P. urinaria*		[141]
147	Nirtetralin	*P. niruri*	Antiviral and hypotensive	[127, 128, 142]
147	Nirtetralin	*P. urinaria*		[125]
147	Nirtetralin	*P. virgatus*	Antiviral	[131, 143]
147	Nirtetralin	*P. amarus*	Anti-inflammatory and antitumor	[132, 144, 145]
148	Nirtetralin A	*P. niruri*	Antiviral	[142]
149	Nirtetralin B	*P. niruri*	Antiviral	[142, 146]
150	Phyllamyricin A	*P. myrtifolius*		[137]
151	Phyllamyricin B	*P. myrtifolius*		[137]
152	Phyllamyricin C	*P. myrtifolius*		[137]
152	Phyllamyricin C	*P. polyphyllus*	Anti-inflammatory	[126]
153	Phyllamyricin D	*P. myrtifolius*		[147]
154	Phyllamyricin E	*P. myrtifolius*		[147]
155	Phyllamyricin F	*P. myrtifolius*		[147]
156	Phyllamyricoside A	*P. myrtifolius*	Anti-HIV	[147]
157	Phyllamyricoside B	*P. myrtifolius*		[147]
158	Phyllamyricoside C	*P. myrtifolius*		[147]
159	Phyllanthostatin A	*P. acuminatus*		[148]
159	Phyllanthostatin A	*P. anisolobus*		[138]
160	Phyllanthuoside C	*P. cochinchinensis*		[149]
161	Phyllanthusmin A	*P. poilanei*		[123]
161	Phyllanthusmin A	*P. oligospermus*	Antitumor	[150]
162	Phyllanthusmin B	*P. reticulatus*		[13]
162	Phyllanthusmin B	*P. poilanei*		[123]
162	Phyllanthusmin B	*P. oligospermus*		[150]
163	Phyllanthusmin C	*P. reticulatus*		[13]
163	Phyllanthusmin C	*P. flexuosus*		[67]
163	Phyllanthusmin C	*P. poilanei*	Antitumor	[123]
163	Phyllanthusmin C	*P. oligospermus*		[150]
164	Phyllanthusmin D	*P. poilanei*		[123]
165	Phyllanthusmin E	*P. poilanei*		[123]
166	Phyllanthusmin D′	*P. flexuosus*		[67]
167	Phyllanthusmin E′	*P. flexuosus*		[67]
168	Phyllanthusmin F	*P. flexuosus*		[67]
169	Phyltetralin	*P. niruri*		[128]
169	Phyltetralin	*P. urinaria*	Anti-inflammatory	[125, 151]
169	Phyltetralin	*P. virgatus*		[131]
169	Phyltetralin	*P. amarus*	Anti-inflammatory	[145]
170	Piscatorin	*P. piscatorum*	Antitumor	[139]
171	Reticulatuside A	*P. reticulatus*		[13]
172	Reticulatuside B	*P. reticulatus*		[13]
173	Retrojusticidin B	*P. myrtifolius*	Anti-HIV	[137, 152]
174	Seco-4-hydroxylintetralin	*P. niruri*		[153]
175	Taxodiifoloside	*P. taxodiifolius*	Antitumor	[124]
176	Urinatetralin	*P. niruri*		[154]
176	Urinatetralin	*P. urinaria*		[125]
177	2,3-Desmethoxy seco-isolintetralin	*P. niruri*		[155]
178	2,3-Desmethoxy seco-isolintetralin diacetate	*P. niruri*		[155]
179	4-(3,4-Dimethoxy-phenyl)-1-(7-methoxy-benzo[1,3]dioxol-5-yl)-2,3-bis-methoxymethyl-butan-1-ol	*P. amarus*		[132]
180	5-Demethoxy niranthin	*P. urinaria*		[125]
180	5-Demethoxy niranthin	*P. amarus*		[132]
181	7′-Hydroxy-3′,4′,5,9,9′-pentamethoxy-3,4-methylene dioxy lignan	*P. urinaria*	Antitumor	[156]
182	Demethylenedioxyniranthin	*P. niruri*		[155]
183	Dihydrocubebin	*P. niruri*		[155]
183	Dihydrocubebin	*P. urinaria*		[73]
184	Hydroxyniranthin	*P. niruri*		[153]
185	Linnanthin	*P. niruri*		[155]
186	Niranthin	*P. niruri*		[157]
186	Niranthin	*P. urinaria*		[125]
186	Niranthin	*P. virgatus*	Antiviral	[131, 143]
186	Niranthin	*P. amarus*	Anti-inflammatory, antiparasitic, antihyperalgesic, and antitumor	[132, 144, 158, 159]
187	Nirphyllin	*P. niruri*		[160]
188	Phyllanthin	*P. niruri*	Hepatoprotection, hypotensive, and antihyperuricemic	[127, 157, 161, 162]
188	Phyllanthin	*P. urinaria*	Immunomodulatory and hypotensive	[125, 130, 163]
188	Phyllanthin	*P. amarus*	Cell-protection, hepatoprotection, antitumor, and anti-CYP3A4	[134, 144, 164, 165]
188	Phyllanthin	*P. fraternus*		[72]
188	Phyllanthin	*P. debilis*		[135]
189	Seco-isolariciresinol	*P. oxyphyllus*		[90]
190	Seco-isolariciresinol trimethyl ether	*P. niruri*		[153]
191	(+)-8-(3,4-(Methylenedioxy)benzyl)-8′-(3′,4′-dimethoxybenzyl)-butyrolactone	*P. virgatus*		[131]
192	(+)-Secoisolariciresinol	*P. songboiensis*		[65]
193	(+)-Songbosin	*P. songboiensis*		[65]
194	2S,3S-Bursehernin	*P. urinaria*		[166]
195	3-(3,4-Dimethoxy-benzyl)-4-(7-methoxy-benzo[1,3]dioxol-5-yl-methyl)-dihydrofuran-2-one	*P. amarus*		[132]
196	Acutissimalignans B	*P. acutissima*		[66]
197	Bursehernin	*P. amarus*		[132]
198	Cubebin dimethyl ether	*P. niruri*		[154]
199	Dibenzylbutyrolactone	*P. niruri*		[153]
200	Heliobuphthalmin lactone	*P. urinaria*		[125]
200	Heliobuphthalmin lactone	*P. amarus*		[132]
201	Hinokinin	*P. niruri*		[136]
201	Hinokinin	*P. virgatus*	Antiviral	[131, 143]
202	(7 R,7′R,8S,8′S)-Icariol A2	*P. urinaria*		[73]
203	Phyllnirurin	*P. niruri*		[160]
204	Urinaligran	*P. urinaria*		[125]
205	Virgatusin	*P. urinaria*		[125]
205	Virgatusin	*P. virgatus*		[131]
205	Virgatusin	*P. amarus*		[132]
206	(+)-Diasyringaresinol	*P. flexuosus*		[67]
207	(−)-Episyringaresinol	*P. urinaria*		[73]
207	(−)-Episyringaresinol	*P. songboiensis*		[65]
208	(−)-Lirioresinol-B	*P. virgatus*		[94]
209	4-Ketopinoresinol	*P. emblica*		[114]
210	4-Oxopinoresinol	*P. urinaria*		[73]
211	Lirioresinol A	*P. emblica*		[114]
212	Medioresinol	*P. emblica*		[114]
213	Pinoresinol	*P. oxyphyllus*		[90]
213	Pinoresinol	*P. songboiensis*		[65]
214	Syringaresinol	*P. emblica*		[114]
214	Syringaresinol	*P. urinaria*		[73]
214	Syringaresinol	*P. reticulatus*		[13]
215	Virgatyne	*P. virgatus*		[94]
216	4,9,9′-Trihydroxy-3,4′-dimethoxy-8-O-3′-neolignan	*P. emblica*		[114]
217	Caffeic acid	*P. urinaria*		[167]
217	Caffeic acid	*P. sellowianus*		[168]
217	Caffeic acid	*P. muellerianus*		[169]
217	Caffeic acid	*P. simplex*		[170]
218	Cinnamic acid	*P. emblica*	Antioxidant	[171]
219	Coniferyl aldehyde	*P. emblica*		[114]
220	Evofolin B	*P. urinaria*		[73]
221	Ferulic acid	*P. urinaria*		[172]
221	Ferulic acid	*P. simplex*		[170]
222	Methyl caffeate	*P. emblica*		[114]
223	Phyllanthuoside A	*P. cochinchinensis*	Antitumor	[149]
224	Phyllanthuoside B	*P. cochinchinensis*		[149]
225	Debelalactone	*P. debilis*	Hepatoprotection	[173]
226	Isofraxidin	*P. sellowianus*		[174]
227	Scopoletin	*P. sellowianus*		[174]
228	1,2,4,6-Tetra-O-galloyl-*β*-D-glucose	*P. emblica*	Antiviral	[175]
228	1,2,4,6-Tetra-O-galloyl-*β*-D-glucose	*P. niruri*	Antiviral	[176, 177]
229	1,3,4,6-Tetra-O-galloyl-*β*-D-glucose	*P. virgatus*		[94]
230	1,4,6-Tri-O-galloyl-*β*-D-glucose	*P. virgatus*		[94]
231	1,6-Di-O-galloyl-*β*-D-glucose	*P. virgatus*		[94]
232	1,2-Di-O-galloyl-3,6-(R)-hexa-hydroxydiphenoyl-*β*-D-glucose	*P. niruri*		[176]
233	Amariin	*P. amarus*	Hepatoprotection, radioprotective, and antioxidant	[178–181]
234	Amariinic acid	*P. amarus*		[182]
235	Amarulone	*P. amarus*		[183]
236	Carpinusnin	*P. emblica*		[184]
237	Chebulagic acid	*P. emblica*	Antioxidant and antitumor	[111, 184, 185]
237	Chebulagic acid	*P. myrtifolius*		[186]
238	Chebulanin	*P. emblica*	Antioxidant	[184, 185]
239	Corilagin	*P. emblica*	Antioxidant and antitumor	[111, 184, 187]
239	Corilagin	*P. niruri*	Antihyperalgesic and anti-inflammatory	[6, 176, 188]
239	Corilagin	*P. urinaria*	Antiviral and antiplatelet	[189–191]
239	Corilagin	*P. reticulatus*		[192]
239	Corilagin	*P. virgatus*		[94]
239	Corilagin	*P. amarus*	Antidiabetic, radioprotective, and anti-HIV	[179, 181, 193, 194]
239	Corilagin	*P. myrtifolius*		[186]
239	Corilagin	*P. muellerianus*		[169]
239	Corilagin	*P. debilis*	Antioxidant	[195]
239	Corilagin	*P. matsumurae*		[196]
239	Corilagin	*P. wightianus*		[89]
239	Corilagin	*P. ussuriensis*	Antioxidant	[197, 198]
240	Excoecarianin	*P. urinaria*	Antiviral	[199]
241	Furosin	*P. emblica*	Antioxidant	[184, 187]
241	Furosin	*P. virgatus*		[94]
241	Furosin	*P. sellowianus*	Antihyperalgesic	[200]
241	Furosin	*P. muellerianus*	Wound healing	[169]
241	Furosin	*P. debilis*	Antioxidant	[195]
242	Geraniin	*P. emblica*	Antioxidant and antitumor	[111, 185, 201]
242	Geraniin	*P. niruri*	Antiviral	[177]
242	Geraniin	*P. urinaria*	Immunomodulatory, antioxidant, and hypotensive	[41, 163]
242	Geraniin	*P. virgatus*	Antiviral	[94, 143]
242	Geraniin	*P. amarus*	Hepatoprotection, radioprotective, and anti-HIV	[179–181, 194]
242	Geraniin	*P. myrtifolius*		[186]
242	Geraniin	*P. sellowianus*	Antihyperalgesic	[200]
242	Geraniin	*P. muellerianus*	Wound healing and antimalarial	[169, 202]
242	Geraniin	*P. debilis*	Antioxidant	[195]
242	Geraniin	*P. matsumurae*		[196]
242	Geraniin	*P. wightianus*		[89]
242	Geraniin	*P. ussuriensis*		[197]
242	Geraniin	*P. caroliniensis*		[203]
243	Geraniinic acid B	*P. amarus*		[182]
244	Hippomanin A	*P. urinaria*	Antiviral	[204]
245	Isocorilagin	*P. emblica*	Antioxidant and antitumor	[185, 201, 205]
245	Isocorilagin	*P. niruri*	Cholinesterase inhibition	[206, 207]
246	Isomallotusinin	*P. emblica*	Antioxidant	[185]
247	Isostrictinin	*P. emblica*		[208]
247	Isostrictinin	*P. urinaria*		[209]
248	Mallonin	*P. emblica*		[184]
249	Mallotusinin	*P. emblica*	Antioxidant	[210]
249	Mallotusinin	*P. myrtifolius*		[186]
250	Neochebulagic acid	*P. emblica*		[184]
251	Phyllanemblinin A	*P. emblica*		[184]
251	Phyllanemblinin A	*P. flexuosus*		[211]
252	Phyllanemblinin B	*P. emblica*		[184]
252	Phyllanemblinin B	*P. flexuosus*		[211]
253	Phyllanemblinin C	*P. emblica*		[184]
253	Phyllanemblinin C	*P. flexuosus*		[211]
254	Phyllanemblinin D	*P. emblica*		[184]
254	Phyllanemblinin D	*P. flexuosus*		[211]
255	Phyllanemblinin E	*P. emblica*		[184]
255	Phyllanemblinin E	*P. flexuosus*		[211]
256	Phyllanemblinin F	*P. emblica*		[184]
257	Phyllanthunin	*P. emblica*		[212]
258	PhyllanthusiinC	*P. myrtifolius*		[186]
259	PhyllanthusiinD	*P. niruri*		[176]
259	PhyllanthusiinD	*P. amarus*	Radioprotective and antioxidant	[178, 181]
260	Phyllanthusiin G	*P. urinaria*		[213]
261	Phyllanthusiin U	*P. urinaria*		[167]
262	Pinocembrin-7-O-[3′′-O-galloyl-4′′,6′′-(S)-hexahydroxydiphenoyl]-*β*-D-glucose	*P. tenellus*		[214]
263	Pinocembrin-7-O-[4′′,6′′-(S)-hexahydroxydiphenoyl]-*β*-D-glucose	*P. tenellus*		[214]
264	Punicafolin	*P. emblica*		[184]
265	Putranjivain A	*P. emblica*		[184]
266	Putranjivain B	*P. emblica*		[185]
267	Repandusinic acid	*P. amarus*	Antioxidant	[178, 182]
268	Terchebin	*P. niruri*		[176]
269	Tercatain	*P. emblica*		[184]
270	Virganin	*P. virgatus*		[94]
271	Dimeric procyanidins mono-gallates	*P. orbicularis*	Antiviral	[53]
272	Dimeric procyanidins-3,3′-di-O-gallates	*P. orbicularis*	Antiviral	[53]
273	Epicatechin-(4*β* → 8)-epigallocatechin	*P. emblica*		[184]
274	Oligomeric procyanidins	*P. orbicularis*	Antiviral	[53]
275	Oligomeric procyanidins mono-gallates	*P. orbicularis*	Antiviral	[53]
276	Phyllemtannin	*P. emblica*	Antitumor	[111]
277	Prodelphinidin B1	*P. emblica*		[184]
277	Prodelphinidin B1	*P. niruri*		[215]
277	Prodelphinidin B1	*P. sellowianus*		[216]
277	Prodelphinidin B1	*P. orbicularis*		[215]
277	Prodelphinidin B1	*P. matsumurae*		[217]
278	Prodelphinidin B2	*P. emblica*		[184]
278	Prodelphinidin B2	*P. orbicularis*	Antioxidant	[53, 54]
278	Prodelphinidin B2	*P. simplex*		[170]
278	Prodelphinidin B2	*P. matsumurae*		[218]
279	Prodelphinidin B-2,3′-O-gallate	*P. emblica*		[184]
280	5,7-Dihydroxy-4′-methoxyflavonol	*P. virgatus*		[94]
281	5,3′-Dihydroxy-6,7,4′-trimethoxyflavone	*P. niruri*		[207]
282	Astragalin	*P. urinaria*		[141]
282	Astragalin	*P. virgatus*		[94]
282	Astragalin	*P. muellerianus*		[169]
283	Avicularin	*P. emblica*		[219]
284	Galangin 3-O-*β*-D-glucoside 8-sulfonate	*P. virgatus*		[94]
285	Isoquercitrin	*P. emblica*		[201]
285	Isoquercitrin	*P. urinaria*		[220]
285	Isoquercitrin	*P. reticulatus*		[192]
285	Isoquercitrin	*P. virgatus*		[94]
285	Isoquercitrin	*P. muellerianus*		[169]
286	Kaempferol	*P. emblica*	Antioxidant	[201]
286	Kaempferol	*P. niruri*		[79]
286	Kaempferol	*P. virgatus*		[94]
286	Kaempferol	*P. cochinchinensis*		[149]
287	Kaempferol-3-O-*α*-L-(6′′-ethyl)-rhamnopyranoside	*P. emblica*		[221]
288	Kaempferol-3-O-*α*-L-(6′′-methyl)-rhamnopyranoside	*P. emblica*		[221]
289	Kaempferol-3-O-*β*-D-glucopyranoside	*P. emblica*	Antioxidant	[201]
290	Kaempferol 8-sulfonate	*P. virgatus*		[94]
291	Myricitrin	*P. virgatus*		[94]
292	Quercetin	*P. emblica*	Antioxidant	[171]
292	Quercetin	*P. urinaria*		[215]
292	Quercetin	*P. virgatus*		[94]
292	Quercetin	*P. caroliniensis*	Anti-inflammatory	[203]
293	Quercetin 3-O-*α*-L-(2,4-di-O-acetyl) rhamnopyranoside-7-O-*α*-L-rhamnopyranoside	*P. urinaria*		[222]
294	Quercetin 3-O-*α*-L-(3,4-di-O-acetyl) rhamnopyranoside-7-O-*α*-L-rhamnopyranoside	*P. urinaria*		[222]
295	Quercetin 3-O-*α*-L-rhamnopyranoside	*P. urinaria*		[222]
296	Quercetin-3-O-*β*-D-glucopyranoside	*P. emblica*	Antioxidant	[201]
297	Quercetin-3-O-*β*-D-glucopyranosyl(1 → 4)-*α*-rhamnopyranoside	*P. niruri*		[79]
298	Quercetin-3-O-*β*-D-glucosyl-(1 → 6)-*β*-D-glucoside	*P. virgatus*		[94]
299	Quercetin 3-O-*β*-D-glucopyranosyl-(2 → 1)-O-*β*-D-xylopyranoside	*P. niruri*		[223]
300	Quercetin pentaacetate	*P. orbicularis*		[54]
301	Quercitrin	*P. niruri*	Antinociceptive	[215, 224]
301	Quercitrin	*P. urinaria*	Anti-inflammatory	[151, 215]
301	Quercitrin	*P. virgatus*		[94]
301	Quercitrin	*P. sellowianus*		[95]
301	Quercitrin	*P. muellerianus*		[169]
301	Quercitrin	*P. orbicularis*		[54]
301	Quercitrin	*P. ussuriensis*		[225]
302	Rhamnocitrin	*P. urinaria*	Anti-inflammatory	[151]
302	Rhamnocitrin	*P. amarus*		[179]
302	Rhamnocitrin	*P. cochinchinensis*		[149]
302	Rhamnocitrin	*P. simplex*		[170]
303	Rutin	*P. niruri*	Anti-inflammatory	[224]
303	Rutin	*P. urinaria*	Anti-inflammatory	[151, 215]
303	Rutin	*P. reticulatus*		[192]
303	Rutin	*P. virgatus*		[94]
303	Rutin	*P. amarus*	Radioprotective and antioxidant	[178, 181]
303	Rutin	*P. debilis*	Antioxidant	[195]
304	Rutin decaacetate	*P. orbicularis*		[54]
305	Schaftoside	*P. cochinchinensis*		[149]
306	Sodium galangin-8-sulfonate	*P. virgatus*		[94]
307	Sodium galangin-3-O-*β*-glucoside-8-sulfonate	*P. virgatus*		[94]
308	Sodium kaempferol-8-sulfonate	*P. virgatus*		[94]
309	Vicenin-2	*P. cochinchinensis*		[149]
310	4′-Methoxyscutellarein	*P. urinaria*		[226]
311	Apigenin	*P. amarus*		[74]
311	Apigenin	*P. orbicularis*	Antioxidant	[54]
312	Apigenin-7-O-(6′′-butyryl-*β*-glucopyranoside)	*P. emblica*		[227]
312	Apigenin-7-O-(6′′-butyryl-*β*-glucopyranoside)	*P. niruri*		[215]
312	Apigenin-7-O-(6′′-butyryl-*β*-glucopyranoside)	*P. urinaria*		[215]
313	Demethoxysudachitin (4′,5,7-trihydroxy-6,8-dimethoxyflavone)	*P. atropurpureus*		[228]
314	Galangin 8-sulfonate	*P. virgatus*		[94]
315	Luteolin	*P. amarus*		[74]
315	Luteolin	*P. singampattiana*		[78]
316	Niruriflavone	*P. niruri*	Antioxidant	[206]
317	Urinariaflavone	*P. urinaria*		[141]
318	2-(4-Hydroxyphenyl)-8-(3-methylbut-2-enyl)-chroman-4-one	*P. niruri*		[23]
319	7-Hydroxyflavanone	*P. sellowianus*		[168]
320	8-(3-Methyl-but-2-enyl)-2-phenyl chroman-4-one	*P. niruri*	Antiparasitic	[23]
321	Nirurin	*P. niruri*		[229]
322	Nirurinetin	*P. niruri*		[229]
323	(S)-Eriodictyol 7-O-(6′′-O-(E)-*β*-coumaroyl)-*β*-D-glucopyranoside	*P. emblica*		[230]
324	(S)-Eriodictyol 7-O-(6′′-O-galloyl)-*β*-D-glucopyranoside	*P. emblica*		[230]
325	(+)-Catechin	*P. niruri*		[176]
325	(+)-Catechin	*P. orbicularis*		[53]
326	(−)-Epiafzelechin	*P. emblica*		[184]
327	(−)-Epicatechin	*P. emblica*		[184]
327	(−)-Epicatechin	*P. niruri*		[176]
327	(−)-Epicatechin	*P. cochinchinensis*		[149]
327	(−)-Epicatechin	*P. orbicularis*		[53]
328	(−)-Epigallocatechin	*P. emblica*		[184]
328	(−)-Epigallocatechin	*P. niruri*		[176]
328	(−)-Epigallocatechin	*P. reticulatus*		[118]
329	(+)-Gallocatechin	*P. emblica*		[184]
329	(+)-Gallocatechin	*P. niruri*		[176]
330	8-(2-Pyrrolidinone-5-yl)-(−)-epicatechin	*P. cochinchinensis*		[149]
331	5,7-Dimethoxy-3,4′-dihydroxy-3′,8-di-C-prenylflavanone	*P. niruri*		[231]
332	5,6,8,4′-Tetrahydroxy isoflavone	*P. atropurpureus*		[228]
333	6-Hydroxy-7,8,2′,3′,4′-pentamethoxyisoflavone	*P. niruri*		[207]
334	(−)-*β*-Sitosterol-3-O-*β*-D-(6-O-palmitoyl) glucopyranoside	*P. songboiensis*		[65]
335	(3*β*,22E)-Stigmasta-5,22-diene-3,25-diol	*P. urinaria*		[73]
336	24-Isopropylcholesterol	*P. niruri*		[157]
337	5*α*,6*β*-Dihydroxysitosterol	*P. emblica*		[232]
338	5*α*,6*β*,7*α*-Trihydroxysitosterol	*P. emblica*		[232]
339	6′-(Stigmast-5-en-3-O-*β*-D-glucopyranosidyl) hexadecanoate	*P. emblica*		[232]
340	6′-(Stigmast-5-en-7-one-3-O-*β*-D-glucopyranosidyl) hexadecanoate	*P. emblica*		[232]
341	7-Ketositosterol	*P. emblica*		[232]
342	7*α*-Hydroxysitosterol	*P. emblica*		[232]
343	7*α*-Acetoxysitosterol	*P. emblica*		[232]
344	7*β*-Ethoxysiterol	*P. emblica*		[232]
345	Amarosterol A	*P. amarus*		[233]
346	Amarosterol B	*P. amarus*		[233]
347	Campesterol	*P. sellowianus*		[216]
348	Daucosterol	*P. emblica*		[232]
348	Daucosterol	*P. urinaria*		[220]
348	Daucosterol	*P. amarus*		[74]
349	Fraternusterol	*P. fraternus*		[234]
350	Phyllanthosecosteryl ester	*P. fraternus*		[234]
351	Phyllanthosterol	*P. fraternus*		[234]
352	Phyllanthostigmasterol	*P. fraternus*		[234]
353	Stigmast-4-en-3-one	*P. emblica*		[232]
354	Stigmast-4-en-3,6-dione	*P. emblica*		[232]
355	Stigmast-4-en-6*β*-ol-3-one	*P. emblica*		[232]
356	Stigmast-4-ene-3*β*,6*α*-diol	*P. emblica*		[232]
357	Stigmast-4,5-en-3-one	*P. oxyphyllus*		[90]
358	Stigmast-5-en-3-ol, oleate	*P. amarus*		[74]
359	Stigmasterol	*P. urinaria*		[97]
359	Stigmasterol	*P. sellowianus*		[216]
359	Stigmasterol	*P. columnaris*		[76]
360	Stigmasterol 3-O-*β*-D-glucoside	*P. urinaria*		[97]
361	*β*-Daucosterol	*P. emblica*	Antioxidant	[171, 212]
362	*β*-Sitosterol	*P. emblica*		[100]
362	*β*-Sitosterol	*P. niruri*		[157]
362	*β*-Sitosterol	*P. urinaria*		[220]
362	*β*-Sitosterol	*P. reticulatus*		[77]
362	*β*-Sitosterol	*P. sellowianus*		[216]
362	*β*-Sitosterol	*P. muellerianus*		[92]
362	*β*-Sitosterol	*P. oxyphyllus*		[90]
362	*β*-Sitosterol	*P. fraternus*		[72]
362	*β*-Sitosterol	*P. debilis*		[135]
362	*β*-Sitosterol	*P. singampattiana*		[78]
363	*β*-Sitosterol-3-O-*β*-D-glucopyranoside	*P. urinaria*		[151]
364	14,15-Dihydroallosecurinin-15*β*-ol	*P. discoideus*		[148]
365	4-Hydroxysecurinine	*P. niruri*		[235]
366	4-Methoxydihydronorsecurinine	*P. niruri*		[235]
367	*β*-Sitosterol-3-*β*-D-glucopyranoside	*P. singampattiana*		[78]
368	4-Methoxynorsecurinine	*P. niruri*		[236]
369	4-Methoxytetrahydrosecurinine	*P. niruri*		[235]
370	Allosecurinine	*P. niruri*		[235]
370	Allosecurinine	*P. glaucus*		[237]
371	Dihydrosecurinine	*P. niruri*		[235]
372	Ent-norsecurinine	*P. niruri*		[238]
373	Epibubbialine	*P. niruri*		[239]
373	Epibubbialine	*P. amarus*		[240]
374	Isobubbialine	*P. niruri*		[215]
374	Isobubbialine	*P. urinaria*		[215]
374	Isobubbialine	*P. amarus*		[240]
375	Methyl (2S)-1-[2-(furan-2-yl)-2-oxoethyl]-5-oxopyrrolidine-2-carboxylate	*P. emblica*		[114]
376	Nirurine	*P. niruri*		[241]
377	Niruroidine	*P. niruroides*		[242]
378	Nitidine	*P. sellowianus*		[243]
379	Norsecurinine	*P. niruri*		[235]
379	Norsecurinine	*P. amarus*	Antifungal	[240, 244]
379	Norsecurinine	*P. simplex*		[245]
379	Norsecurinine	*P. discoides*		[246]
380	Phyllanthine	*P. niruri*		[236]
380	Phyllanthine	*P. amarus*		[240]
381	Securinine	*P. niruri*		[235]
381	Securinine	*P. amarus*		[240]
381	Securinine	*P. glaucus*		[237]
382	Securinol A	*P. niruri*		[235]
383	Securinol B	*P. niruri*		[235]
384	Simplexine	*P. simplex*		[245]
385	Tetrahydrosecurinine	*P. niruri*		[235]
386	Virosecurinine	*P. discoides*		[247]
387	1,12-Diazacyclodocosane-2,11-dione	*P. niruri*		[248]
388	3-(3-Methylbut-2-en-1-yl) isoguanine	*P. reticulatus*		[118]
389	5-Hydroxy-isoquinoline	*P. emblica*		[249]
390	E,E-2,4-Octadienamide	*P. fraternus*	Antimalarial	[250]
391	E,Z-2,4-Decadienamide	*P. fraternus*	Antimalarial	[250]
392	Indole-3-carboxaldehyde	*P. virgatus*		[94]
393	Indole-3-carboxylic acid	*P. virgatus*		[131]
394	Phyllanthimide	*P. sellowianus*		[251]
395	Phyllurine	*P. urinaria*		[252]
396	(−)-Epicatechin 3-O-gallate	*P. niruri*		[176]
396	(−)-Epicatechin 3-O-gallate	*P. orbicularis*	Antiviral	[53]
397	(−)-Epigallocatechin 3-O-gallate	*P. emblica*		[111]
397	(−)-Epigallocatechin 3-O-gallate	*P. niruri*		[176]
398	(5R^*∗*^R^*∗*^)-4,6-Dimethoxycarbonyl-5-[2′,3′,4′-trihydroxy-6′-(methoxycarbonyl) phenyl]-5,6-dihydro-2H-pyran-2-one	*P. reticulatus*		[16]
399	1-O-Galloyl-6-O-luteoyl-*α*-D-glucose	*P. niruri*	Antimalarial	[223]
400	1-O-Galloyl-*β*-D-glucose	*P. emblica*	Antidiabetic and antitumor	[111, 253, 254]
400	1-O-Galloyl-*β*-D-glucose	*P. virgatus*		[94]
401	2-(2-Methylbutyryl)phloroglucinol 1-O-(6′′-O-*β*-D-apiofuranosyl)-*β*-D-glucopyranoside	*P. emblica*		[230]
402	2,3,4,5,6-Pentahydroxybenzoic acid	*P. urinaria*		[255]
403	2,3,5,6-Tetrahydroxybenzyl acetate	*P. niruri*		[256]
404	2,6-Dimethoxy-4-(2-hydroxyethyl)phenol 1-O-*β*-D-glucopyranoside	*P. emblica*		[110]
405	2-Carboxylmethylphenol 1-O-*β*-D-glucopyranoside	*P. emblica*		[110]
406	3′′-Hydroxy robustaside A (6′-(3′′,4′′-dihydroxy cinnamoyl) arbutin)	*P. atropurpureus*		[228]
407	3,3′-Di-O-methylellagic acid	*P. reticulatus*		[105]
408	3,4,3′-Tri-O-methylellagic acid	*P. urinaria*		[172]
408	3,4,3′-Tri-O-methylellagic acid	*P. reticulatus*		[16]
409	3,4,8,9,10-Pentahydroxy-dibenzo[b,d] pyran-6-one	*P. emblica*		[114]
410	3,4-di-O-Methylellagic acid	*P. reticulatus*		[105]
411	3,5-Dicaffeoylquinic acid	*P. muellerianus*		[169]
412	3,5-Dihydroxy-4-methoxybenzoic acid	*P. urinaria*		[73]
413	3-Ethylgallic acid	*P. emblica*		[208]
414	3-O-Methylellagic acid 4′-O-*α*-L-rhamnopyranoside	*P. reticulatus*		[105]
415	4,4′-Di-O-methylellagic acid	*P. reticulatus*		[105]
416	4-Hydroxy-3-methoxybenzaldehyde	*P. emblica*		[114]
417	4-Hydroxy-3-methoxy-benzoic acid	*P. amarus*		[74]
418	4-O-Caffeoylquinic acid	*P. niruri*		[257]
419	4-O-Methylellagic acid-3′-*α*-rhamnoside	*P. emblica*		[87]
420	4-O-Methylgallic acid	*P. polyphyllus*	Anti-inflammatory	[126]
421	8,9-Epoxy brevifolin	*P. simplex*	Hepatoprotective	[258]
422	Bergenin	*P. flexuosus*		[80]
422	Bergenin	*P. wightianus*		[89]
423	Brevifolin	*P. urinaria*		[259]
423	Brevifolin	*P. virgatus*		[94]
423	Brevifolin	*P. simplex*	Hepatoprotective	[260]
424	Brevifolin carboxylic acid	*P. niruri*		[261]
424	Brevifolin carboxylic acid	*P. urinaria*		[209]
424	Brevifolin carboxylic acid	*P. amarus*	Antidiabetic	[193]
424	Brevifolin carboxylic acid	*P. matsumurae*		[196]
425	Caffeoylmalic acid	*P. muellerianus*		[169]
426	Chebulic acid	*P. emblica*		[253]
427	Chlorogenic acid	*P. sellowianus*		[168]
427	Chlorogenic acid	*P. muellerianus*		[169]
428	Dehydrochebulic acid trimethyl ester	*P. urinaria*		[73]
429	Di [3,4,5-trihydroxy-phenyl] ether	*P. atropurpureus*		[228]
430	Ellagic acid	*P. emblica*	Antioxidant	[100, 210]
430	Ellagic acid	*P. niruri*	Antidiabetic	[202, 261]
430	Ellagic acid	*P. urinaria*	Antitumor	[220, 262]
430	Ellagic acid	*P. reticulatus*		[192]
430	Ellagic acid	*P. matsumurae*		[196]
430	Ellagic acid	*P. wightianus*		[89]
431	Ethyl brevifolin carboxylate	*P. niruri*		[261]
431	Ethyl brevifolin carboxylate	*P. urinaria*		[189]
432	Ethyl gallate	*P. emblica*	Antitussive	[212, 263]
432	Ethyl gallate	*P. myrtifolius*		[186]
433	Flavogallonic acid bislactone	*P. emblica*		[184]
434	Gallic acid	*P. emblica*	Antiulcer and antioxidant	[210, 264]
434	Gallic acid	*P. niruri*	Anti-inflammatory	[202, 224]
434	Gallic acid	*P. urinaria*		[220]
434	Gallic acid	*P. virgatus*		[94]
434	Gallic acid	*P. amarus*	Antijaundice	[265]
434	Gallic acid	*P. myrtifolius*		[186]
434	Gallic acid	*P. muellerianus*		[169]
434	Gallic acid	*P. debilis*	Antioxidant	[195]
434	Gallic acid	*P. simplex*		[170]
434	Gallic acid	*P. matsumurae*		[196]
434	Gallic acid	*P. wightianus*		[89]
434	Gallic acid	*P. ussuriensis*		[225]
435	Gallic acid 3-O-(6′-O-galloyl)-*β*-D-glucoside	*P. emblica*		[184]
436	Gallic acid 3-O-*β*-D-glucoside	*P. emblica*		[184]
437	Gallic acid 4-methyl ether	*P. cochinchinensis*		[149]
438	Gallic acid ethyl ester	*P. urinaria*	Antihyperalgesic	[266]
438	Gallic acid ethyl ester	*P. sellowianus*		[95]
438	Gallic acid ethyl ester	*P. caroliniensis*	Anti-inflammatory	[203]
439	Koaburaside	*P. cochinchinensis*		[149]
440	L-Malic acid 2-O-gallate	*P. emblica*	Antitumor	[111, 253]
441	Methyl-4-hydroxybenzoate	*P. emblica*		[114]
442	Methyl brevifolin carboxylate	*P. niruri*	Hypotensive and antiplatelet	[206, 267, 268]
442	Methyl brevifolin carboxylate	*P. urinaria*	Antioxidant and anti-inflammatory	[151, 269]
442	Methyl brevifolin carboxylate	*P. reticulatus*		[192]
442	Methyl brevifolin carboxylate	*P. virgatus*		[94]
443	Methyl ester dehydrochebulic acid	*P. urinaria*		[269]
444	Methyl gallate	*P. emblica*	Antioxidant and antitussive	[187, 263]
444	Methyl gallate	*P. urinaria*	Antioxidant and anti-inflammatory	[151]
444	Methyl gallate	*P. reticulatus*		[192]
444	Methyl gallate	*P. virgatus*		[94]
444	Methyl gallate	*P. myrtifolius*		[186]
444	Methyl gallate	*P. muellerianus*		[169]
444	Methyl gallate	*P. ussuriensis*		[197]
445	Mucic acid 1,4-lactone 2-O-gallate	*P. emblica*		[253]
446	Mucic acid 1,4-lactone 3,5-di-O-gallate	*P. emblica*		[253]
447	Mucic acid 1,4-lactone 3-O-gallate	*P. emblica*	Antioxidant	[185, 253]
448	Mucic acid 1,4-lactone 5-O-gallate	*P. emblica*		[253]
449	Mucic acid 1,4-lactone 6-methyl ester 2-O-gallate	*P. emblica*		[253]
450	Mucic acid 1,4-lactone 6-methyl ester 5-O-gallate	*P. emblica*		[253]
451	Mucic acid 1-methyl ester 2-O-gallate	*P. emblica*		[253]
452	Mucic acid 2-O-gallate	*P. emblica*	Antitumor	[111, 253]
453	Mucic acid 3-O-gallate	*P. emblica*		[270]
454	Mucic acid 6-methyl ester 2-O-gallate	*P. emblica*		[253]
455	Mucic acid di-methyl ester 2-O-gallate	*P. emblica*		[253]
456	p-Hydroxybenzaldehyde	*P. urinaria*		[73]
457	Phloroglucinol	*P. ussuriensis*		[225]
458	Phyllangin	*P. niruri*		[256]
459	Phyllanthusin F	*P. urinaria*		[271]
460	Potassium brevifolin carboxylate	*P. virgatus*		[94]
461	Protocatechuic acid	*P. urinaria*		[189]
461	Protocatechuic acid	*P. matsumurae*		[196]
462	Pyrogallol	*P. emblica*	Antitumor and anti-inflammatory	[249, 272]
462	Pyrogallol	*P. urinaria*		[167]
463	Robustaside A	*P. atropurpureus*	Antitumor	[228]
464	Shikimic acid	*P. myrtifolius*		[186]
465	Syringaldehyde	*P. emblica*		[114]
466	Tri-Me dehydrochebulic acid	*P. urinaria*		[220]
467	Trimethyl-3,4-dehydrochebulate	*P. urinaria*	Antioxidant and anti-inflammatory	[151]
468	Vanillic acid	*P. emblica*		[114]
469	(−)-7′-Hydroxydivanillyltetrahydrofuran	*P. songboiensis*		[65]
470	(+)-Cucurbic acid	*P. urinaria*		[73]
471	(+)-Methyl cucurbate	*P. urinaria*		[73]
472	(E)-3-(5′-Hydroperoxy-2,2′-dihydroxy[1,1′-biphenyl]-4-yl)-2-propenoic acid	*P. urinaria*		[255]
473	1′S-11-Dehydroxy penicillide	*P. emblica*		[114]
474	2R-Diethyl malate	*P. emblica*		[114]
475	3,6′-Di-O-benzoyl-2′-O-acetylsucrose	*P. cochinchinensis*		[108]
476	3,6′-Di-O-benzoyl-3′-O-acetylsucrose	*P. cochinchinensis*		[108]
477	3,6′-Di-O-benzoyl-4′-O-acetylsucrose	*P. cochinchinensis*		[108]
478	3,6′-Di-O-benzoylsucrose	*P. cochinchinensis*		[108]
479	3,4-Dimethoxyphenyl-*β*-D-glucopyranoside	*P. cochinchinensis*		[149]
480	3,4-Dihydroxyphenylpropanol 3-O-*β*-D-glucopyranoside	*P. reticulatus*		[118]
481	3,4,5-Trimethoxy-phenyl-*β*-D-glucopyranoside	*P. cochinchinensis*		[149]
482	3-O-Benzoyl-6′-O-(E)-cinnamoylsucrose	*P. cochinchinensis*		[108]
483	4,4,8-Trimethoxy chroman	*P. amarus*		[273]
484	5-Hydroxymethyl-2-furaldehyde	*P. urinaria*		[73]
485	4-Hydroxysesamin	*P. niruri*		[274]
486	5-Hydroxymethylfurfural	*P. emblica*	Antioxidant	[171]
487	Aquilegiolide	*P. anisolobus*		[138]
487	Aquilegiolide	*P. klotzschianus*		[275]
488	Bis(2-ethylicosyl)phthalate	*P. muellerianus*		[92]
489	Bis(2-ethyloctyl)phthalate	*P. muellerianus*		[92]
490	Di-O-methylcrenatin	*P. cochinchinensis*		[149]
491	Byzantionoside B	*P. multiflorus*		[276]
492	Carthamoside B5	*P. reticulatus*		[118]
493	Dendranthemoside B	*P. urinaria*		[141]
494	Hovetrichoside A	*P. reticulatus*		[118]
495	Isotachioside	*P. reticulatus*		[118]
496	Menisdaurilide	*P. anisolobus*		[138]
496	Menisdaurilide	*P. klotzschianus*		[275]
497	Methyl (1 R,2R,2′Z)-2-(5′-hydroxy-pent-2′-enyl)-3-oxocyclopentaneacetate	*P. urinaria*		[73]
498	Mucic acid	*P. emblica*		[277]
499	Mucic acid 1-methyl ester-6-ethyl ester	*P. emblica*		[114]
500	Penicillide	*P. emblica*		[114]
501	Phthalic acid bis(2,5-dimethylhexyl) ester	*P. urinaria*		[99]
502	Phyllanthoid A	*P. cochinchinensis*	Antitumor	[278]
503	Phyllanthoid B	*P. cochinchinensis*		[278]
504	Phyllanthurinolactone	*P. urinaria*		[279]
505	Phyllanthusone	*P. fraternus*		[121]
506	Phyllester	*P. niruri*		[157]
507	Purpactin A	*P. emblica*		[114]
508	Roseoside	*P. multiflorus*		[276]
509	Succinic acid	*P. niruri*		[280]
510	Terephthalic acid mono-[2-(4-carboxy-phenoxycarbonyl)-vinyl] ester	*P. urinaria*		[255]
511	Vanilloloside	*P. cochinchinensis*		[149]
512	Xanthoxyline	*P. sellowianus*		[281]
